# A typical canine Ehlers-Danlos-like syndrome without collagen abnormalities: a suspected case of Tenascin-X deficiency

**DOI:** 10.1186/s12917-025-05271-0

**Published:** 2026-01-17

**Authors:** Belén M. Rivera Gomez-Barris, Carlos González, Constanza Toro-Valdivieso

**Affiliations:** 1 Clínica Veterinaria Oftaderm, Oftalmología y Dermatología Veterinaria, Av. Francisco de Bilbao 7341, 7850000 Santiago, Chile; 2 Facultad de Medicina Veterinaria, Universidad Andrés Bello, República 440, 8320000 Santiago, Chile; 3 Department of Veterinary Medicine, University of Cambridge, Cambridge, UK

**Keywords:** Ehlers-Danlos syndrome, Canine, Spontaneous evisceration, Vascular fragility, Elastic fibers, Tenascin X

## Abstract

**Background:**

Ehlers-Danlos syndromes (EDS) are rare heritable connective tissue disorders, most commonly linked to collagen abnormalities. In dogs, reported cases are infrequent and typically involve skin fragility and joint laxity, with limited understanding of underlying genetic causes. This report describes an unusual, aggressive, and fatal case of an Ehlers–Danlos–like syndrome (EDlS) in a Maltese dog, with several uncommon features. Unlike most canine EDS cases, which show collagen defects, this case revealed minimal collagen alterations; instead, elastic fibers were primarily affected.

**Case report:**

A one-and-a-half-year-old male Maltese dog presented with progressive abdominal masses, skin fragility, joint deformities, and frequent bruising since early life. Clinical examination revealed hyperextensible, fragile skin, hematomas, and contractures of the hind limbs. Imaging confirmed a hernia lacking supportive connective structures. Histopathological analysis showed elastic fiber hypertrophy and fragmentation, with minimal collagen changes. Despite palliative wound management, the patient died within ten days of the initial consultation due to spontaneous evisceration and vascular rupture.

**Conclusion:**

The histological features are consistent with a possible Tenascin-X deficiency. Definitive molecular classification was beyond the scope of this case. This report expands the spectrum of EDlS in dogs.

## Background

Ehlers–Danlos syndromes (EDS) are a group of heritable disorders affecting connective tissues. Depending on the EDS subtype, clinical presentation may include skin hyperextensibility and fragility, spontaneous wounds and bruising, delayed wound healing, joint hypermobility, ocular abnormalities, hernias, and vascular fragility [1].

Although most commonly reported in humans, EDS has also been described in dogs [2–4]. Dermatological manifestations are the most frequent presentation in canine cases, with some dogs also exhibiting joint laxity and ocular abnormalities [2, 3].

EDS can be diagnosed based on clinical history, supported by ancillary tests in which histopathological analysis plays a relevant role. However, genetic testing is required to establish the specific EDS subtype. In humans, 14 EDS subtypes associated with 20 EDS-linked genes have been described [1], and disease severity and clinical expression depend on the affected gene and the nature of the mutation. While Ehlers–Danlos syndromes are well classified in humans, comparable molecular classification in dogs remains limited, and most diagnoses rely on clinical and histopathological features. Unfortunately, genetic testing is rarely performed in canine patients; consequently little is known about EDS-associated genetic variants and their corresponding clinical phenotypes in dogs.

To date, pathogenic variants have been reported in COL5A1 and ADAMTS2 [5–7]. More recently, mutations in the TNXB gene have also been identified in dogs [4]. EDS associated with COL5A1 is characterised by abnormalities in type V collagen, whereas ADAMTS2-related EDS results in defective collagen fibril formation due to impaired procollagen processing [8]. In contrast, Tenascin-X is a large extracellular matrix glycoprotein that plays a crucial role in connective tissue organisation and maintenance [1, 8].

This report describes an unusual and severe case of an Ehlers–Danlos–like syndrome (EDlS) in a Maltese dog. Although genetic testing was not performed, the clinical presentation and histopathological findings suggest involvement of a rare EDS subtype predominantly affecting elastic fibres.

## Case presentation

### Anamnesis

**Fig. 1:**
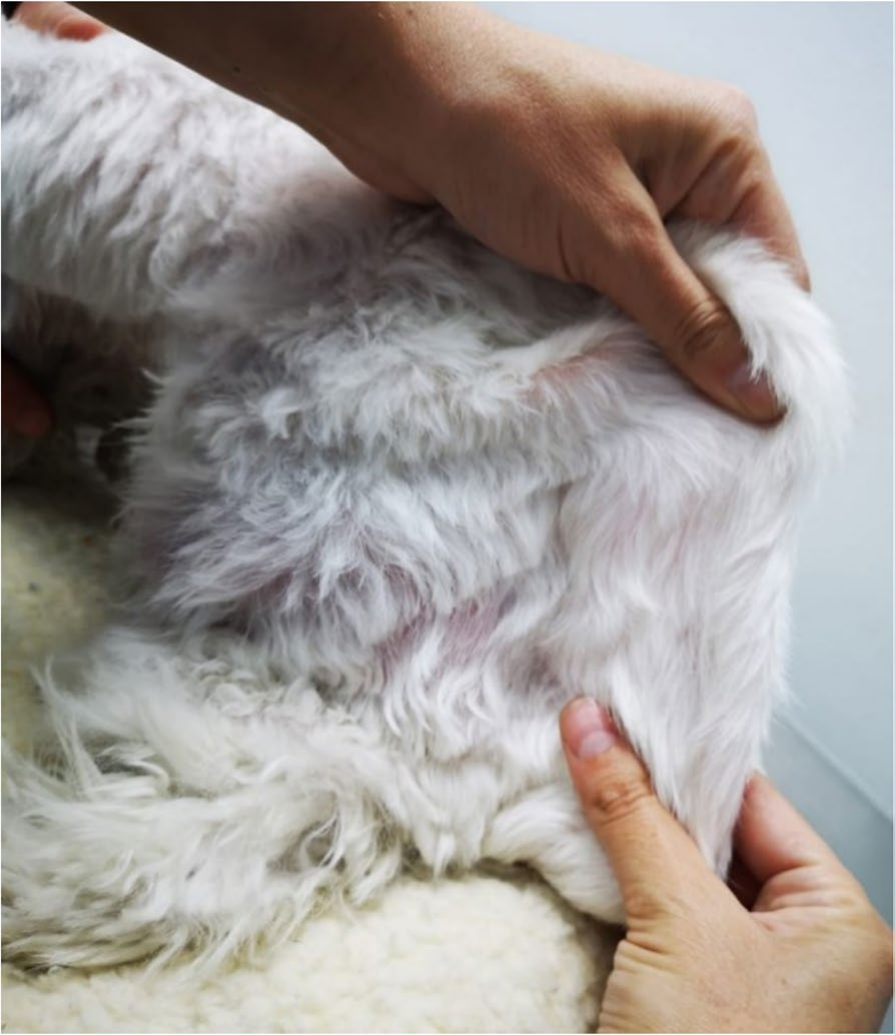
Marked skin hyperextensibility over the dorsum

**Fig. 2:**
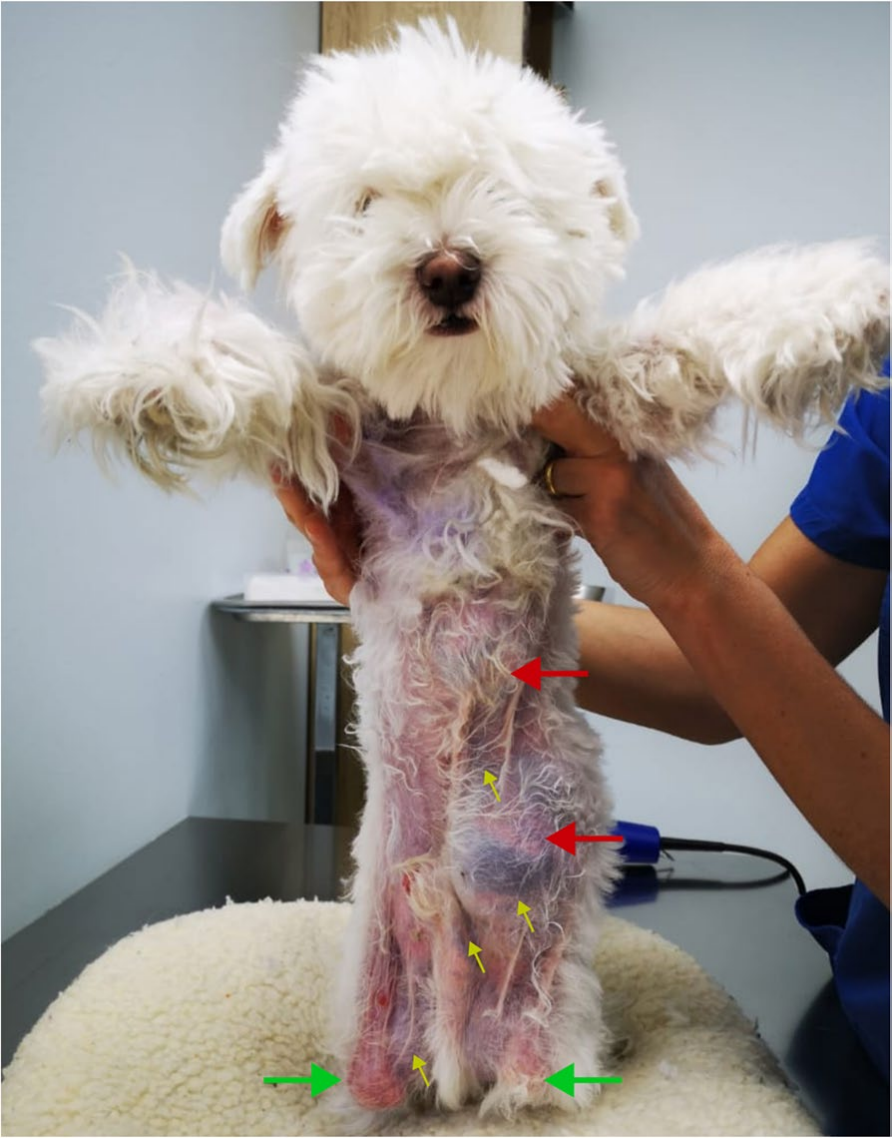
Gross clinical findings. Red arrows show two abdominal masses. Green arrows show bilateral permanent knee flexion contractures. Yellow arrows highlight multiple areas of cutaneous bruising

A one-and-a-half-year-old intact male Maltese dog was presented with an abdominal mass showing progressive growth. Furthermore, skin fragility and hyperflexibility (Fig. 1), as well as frequent bruising, had been observed since acquisition from a non-official breeder at two months of age. The patient was constantly treated by a different practitioner with topical products (Fusidic Acid - Fucidin 2% and Chloramphenicol 3% Spray - Eximerk) twice daily to manage and control wound infections. Additionally, the patient’s hind limbs gradually deformed into a permanent knee flexion contracture severely limiting the dog’s mobility (Fig. 2). When seen by an orthopedic specialist, bilateral posterior muscle and patellar atrophy were found. Corrective surgery was scheduled but was cancelled due to the fragility of both the skin and blood vessels.

### Clinical examination

Despite the evident clinical issues described above, the patient was otherwise clinically stable with normal vital signs. When examining the skin, it was very thin and fragile, exhibiting telangiectasia and several hematomas in various body locations (Figs. 1 and 2). The skin was also hyperflexible with a skin extensibility index (SEI) of 17%. Less than 14.5% SEI is considered normal in dogs. When examining the abdomen, two masses were observed: one on the left side of the lower abdomen and a smaller one at the level of the xiphoid appendix Fig. 2. Upon palpation, the abdominal mass was described as a hernia, while the xiphoid one was a mass of soft consistency of undetermined origin.

### Ancillary tests

**Fig. 3:**
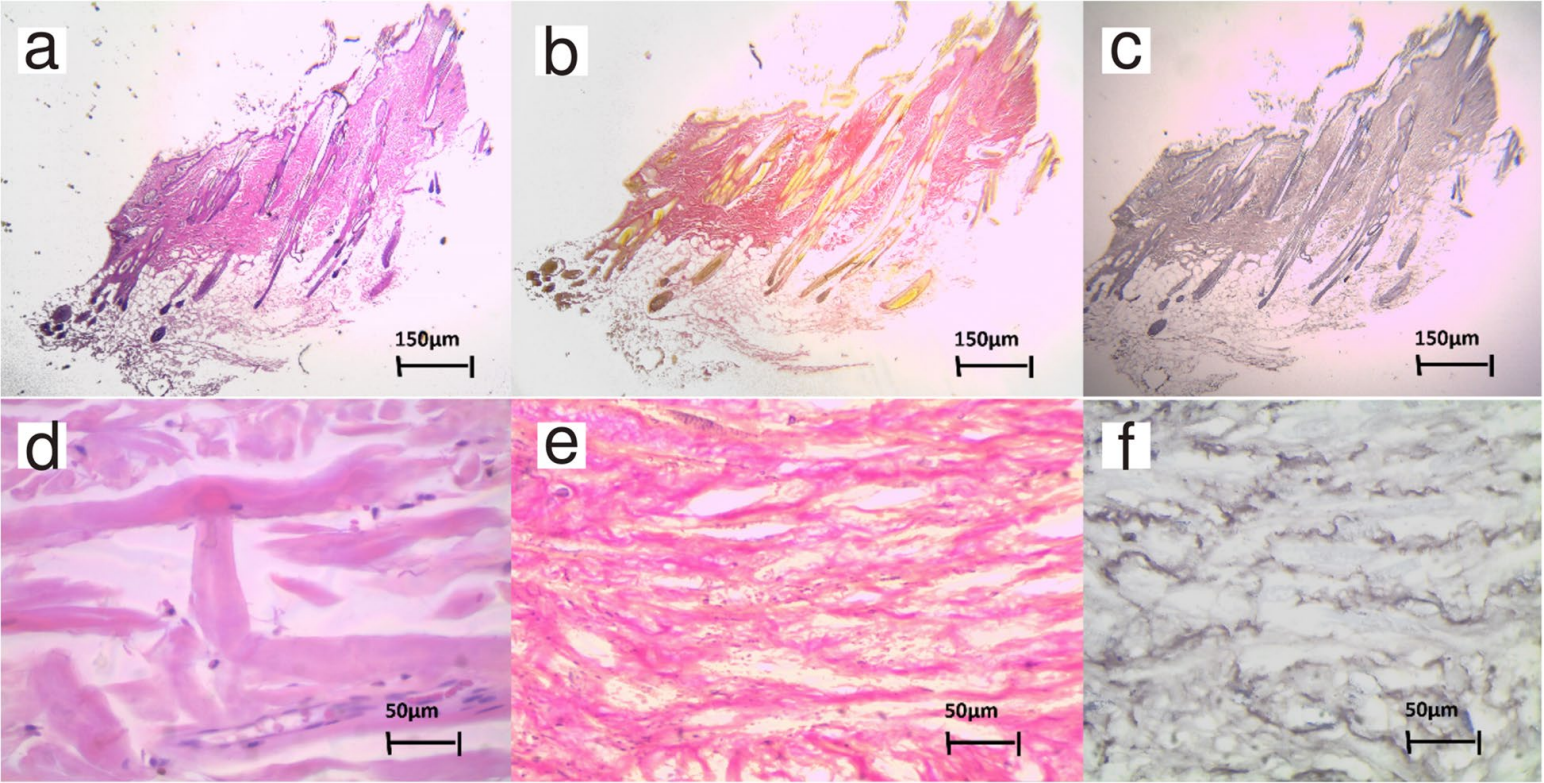
Histopathological evaluation of skin biopsies. Hematoxylin and Eosin staining (**A**, **D**) shows general dermal architecture with mild collagen disorganisation and fragmentation at high magnification (**D**, 400x). Van Gieson staining (**B**, **E**) highlights dense collagen I fibres, which appear largely preserved (**E**, 200x). Orcein staining (**C**, **F**) demonstrates marked elastic fibre abnormalities, including increased number, hypertrophy, fragmentation, and architectural disorganisation of elastic fibres within the dermis (**F**, 200x). Panels **A**–**C** show the same biopsy fragment at low magnification (40x)

Further tests were performed to supplement the information obtained during the anamnesis and clinical examination. No alterations were observed in the hemogram or in the serum biochemical profile. An ultrasound confirmed the presence of a hernia in the left caudal abdomen containing intestinal loops, the left kidney and a segment of the spleen. The hernia showed absence of muscle, aponeurosis and hernial ring, indicating structural weakness of the connective tissues. The smaller xiphoid mass remained undefined. Finally, three skin biopsies were collected and sent for histopathological analysis. Two fragments exhibited a hemorrhagic crust on the epidermal surface. The epidermis appears thinned, with one or at most two layers of keratinocytes, showing apparent dermal atrophy, which may also be merely thinning resulting from marked tissue stretching. Furthermore, there was hypertrophy of the network of elastic fibers in the papillary and deep dermis, much more pronounced in the latter (Fig. 3F), where they form thick bundles that were abruptly interrupted in some areas by the presence of cutaneous appendages. Foci of microhemorrhage were also observed, at the level at which the elastic network is interrupted, with fragmentation of the elastic fibres and marked variation in their diameter. No significant changes were observed in the collagen fibers, apart from some fiber fragmentation and disarrangement (Fig. 2D). Furthermore, the Image ProPlus 4.5 morphometric software-assisted analysis showed a clear increment of the elastin component in the dermis, estimated at 28.3% of the total Collagen and elastic fibrous tissue area. This percentage was higher than the proportion estimated for most animal species and humans, around 12% [9]. These histopathological cutaneous alterations with epidermal atrophy and hypertrophy of elastic tissue were compatible with Ehlers-Danlos syndrome. Based on all the available information, the patient was diagnosed with an EDlS. Furthermore, the histopathological findings, where collagen was almost unaltered, suggest this could be a case of classical-like EDS due to tenascin-X deficiency. However, genetic testing, necessary to confirm the type of EDS, was not authorized by the owners.

### Treatment and progression

There is no cure for these syndromes, and only palliative care can be provided to help manage current issues (e.g. wounds) and prevent further injuries. Prontosan® wound gel was used for wound management. Within ten days of the first consultation, the patient suffered a severe spontaneous hernial rupture. Some major blood vessels were damaged. Whether this vessel damage was spontaneous or caused by the traction of the protruding organs remains undetermined. This hernial complication resulted in a hypovolemic shock and cardiac arrest, leading to the patient’s death. Due to the genetic origin of this disease, the owners were strongly advised to let the breeders know about the syndrome to avoid further cases.

## Discussion

In normal canine dermis, elastic fibres form a fine, evenly distributed network interwoven with collagen bundles, contributing to tissue resilience and recoil. In contrast, the dermis in the present case showed marked elastic fibre hypertrophy, fragmentation, and architectural disorganisation, with only minimal collagen alterations. Previously reported cases of canine Ehlers–Danlos syndrome have predominantly emphasised collagen abnormalities, including disrupted collagen fibril organisation and dermatosparactic changes, as described in association with COL5A1 and ADAMTS2 variants [4, 6, 7, 10, 11]. Elastic fibre abnormalities have been less frequently documented in canine cases, making the histopathological pattern observed here atypical within the current canine EDS literature and more closely aligned with Tenascin-X–related phenotypes described in humans [8].

Ehlers–Danlos syndromes are rarely reported in dogs, and most canine cases are diagnosed based on clinical presentation supported by histopathological findings. This manuscript presents a severe and unusual case of an Ehlers–Danlos–like syndrome in a Maltese dog. Common EDS symptoms previously described in dog cases, such as skin hyperelasticity and fragility were present. However, various aspects of the case presented here were unusual and, thus, worth reporting. For example, although joint hypermobility has been described in canine EDS [12], to the authors’ knowledge the severe permanent knee flexion contracture seen in this case has not been previously reported. Another unusual characteristic of this case was the presence of extremely fragile blood vessels. In dogs, blood vessel fragility is commonly associated with ADAMTS2 and COL5A1 mutations. However, this feature is not well documented in the literature, with only one published EDS case reporting severe vascular fragility [11]. It is possible that the combination of blood vessel fragility and the traction effect during the evisceration process may explain the rupture of major blood vessels observed in the present case.

The histological findings were particularly interesting. Unlike other dog-related cases, there were almost no alteration in collagen or collagen organisation [2, 3, 10, 12]. Instead, alterations were mostly affecting elastic fibers. These findings are consistent with those described in human cases of classical-like EDS, a very rare EDS type linked to variations in the TNXB gene encoding Tenascin-X [1, 8]. Symptoms commonly found in this type of EDS include skin hyperextensibility, generalized joint hypermobility, and easily bruisable skin [1]. Additionally, muscle weakness and distal contractures have also been reported.

Only one publication has reported a possible case of TNXB-linked EDS in a dog [4]. This study focused specifically on the genetic characterisation of the affected individual and identified two rare variants in the TNXB gene, each inherited from a different parent. However, no histopathological findings were described. As this report was based on a single case and lacked functional evidence of TNXB disruption, the diagnosis remains uncertain. In contrast, our case presents histopathological changes strongly suggestive of TNXB-related EDS, although genetic testing was not performed. Given the limited number of reported cases and the lack of functional or genetic confirmation in most, the role of TNXB mutations in canine EDS remains unclear.

Finally, this report is subject to important limitations. Definitive molecular confirmation was not possible, as genetic testing, immunohistochemical characterisation, ultrastructural (electron microscopic) analysis, and post-mortem examination were not performed. Consequently, the diagnosis remains presumptive and based on clinicopathological correlation rather than direct molecular evidence. These limitations reflect common constraints encountered in veterinary clinical practice and should be considered when interpreting the findings.

Acquired dermal remodelling secondary to chronic trauma or inflammation was considered; however, the particular combination of very early onset of clinical signs, severe clinical course, and distinctive elastic fibre pathology supports classification of this case as an EDlS with a phenotype suggestive of Tenascin-X deficiency [1, 8]. This report expands the recognised histopathological spectrum of suspected canine EDS and highlights the potential contribution of elastic fibre pathology in severe connective tissue disease. Further well-documented cases integrating clinical findings, histopathology, and molecular analyses will be essential to clarify genotype–phenotype relationships in canine EDlS [2, 3].

## Conclusion

Overall, the case presented here was particularly severe and resulted in sudden death. While clinical evaluation supported by ancillary tests may raise suspicion of an inherited connective tissue disorder, definitive classification of Ehlers–Danlos syndrome subtypes requires molecular confirmation, which was not available in this case. Nevertheless, the clinical presentation and histopathological findings are most consistent with an Ehlers–Danlos–like syndrome, with a phenotype suggestive of Tenascin-X involvement.

This report expands the recognised clinical and histopathological spectrum of suspected canine EDlS and highlights the importance of early recognition and cautious clinical management in affected dogs.

## Data Availability

No datasets were generated or analysed during the current study.

## Abbreviation

EDS: Ehlers-Danlos

## References

[1] Malfait F, Castori M, Francomano CA, Giunta C, Kosho T, Byers PH. The Ehlers-Danlos syndromes. Nat Rev Dis Primers. 2020;6(1):64. 10.1038/s41572-020-0194-9. 32732924 10.1038/s41572-020-0194-9

[2] Roberts JH, Halper J. Connective tissue disorders in domestic animals. In: Progress in Heritable Soft Connective Tissue Diseases. Springer , Cham, Switzerland ; 2021. pp. 325–35. 10.1007/978-3-030-80614-9_15 34807427

[3] Miller WH, Griffin CE, Campbell KL. Congenital and hereditary defects. In: Muller and Kirk’s Small Animal Dermatology. 7th ed. Elsevier, St. Louis, MO, USA.; 2013. pp. 602–4.

[4] Bauer A, Bateman JF, Lamandé SR, Hanssen E, Kirejczyk SG, Yee M, et al. Identification of two independent COL5A1 variants in dogs with Ehlers-Danlos syndrome. Genes. 2019;10(10):731. 10.3390/genes10100731. 31546637 10.3390/genes10100731 PMC6826881

[5] Bauer A, de Lucia M, Leuthard F, Jagannathan V, Leeb T. Compound heterozygosity for TNXB genetic variants in a mixed-breed dog with Ehlers-Danlos syndrome. Anim Genet. 2019;50(5):546–9. 10.1111/age.12830. 31365140 10.1111/age.12830

[6] Jaffey JA, Bullock G, Teplin E, Guo J, Villani NA, Mhlanga-Mutangadura T, et al. A homozygous ADAMTS2 nonsense mutation in a Doberman Pinscher dog with Ehlers Danlos syndrome and extreme skin fragility. Anim Genet. 2019;50(5):543–5. 10.1111/age.12825. 31294848 10.1111/age.12825 PMC6771693

[7] Jaffey JA, Bullock G, Guo J, Mhlanga-Mutangadura T, O’Brien DP, Coates JR, et al. Novel Homozygous ADAMTS2 Variants and Associated Disease Phenotypes in Dogs with Dermatosparactic Ehlers-Danlos Syndrome. Genes. 2022;13(11):2158. 10.3390/genes13112158. 36421833 10.3390/genes13112158 PMC9690363

[8] Zweers MC, Dean WB, Van Kuppevelt TH, Bristow J, Schalkwijk J. Elastic fiber abnormalities in hypermobility type Ehlers-Danlos syndrome patients with tenascin-X mutations. Clin Genet. 2005;67(4):330–4. 10.1111/j.1399-0004.2005.00401.x. 15733269 10.1111/j.1399-0004.2005.00401.x

[9] Matveeva I, Myakinin O. Monte Carlo modelling of normal skin and skin cancer Raman spectra. In: Journal of Physics: Conference Series. vol. 1368. IOP Publishing; 2019. p. 042084. 10.1088/1742-6596/1368/4/042084.

[10] Paciello OR, Lamagna FR, Lamagna BA, Papparella SE. Ehlers-Danlos–like syndrome in 2 dogs: clinical, histologic, and ultrastructural findings. Vet Clin Pathol. 2003;32(1):13–8. 10.1111/j.1939-165X.2003.tb00306.x. 12655483 10.1111/j.1939-165x.2003.tb00306.x

[11] Uri M, Verin R, Ressel L, Buckley L, McEwan N. Ehlers-Danlos syndrome associated with fatal spontaneous vascular rupture in a dog. J Comp Pathol. 2015;152(2–3):211–6. 10.1016/j.jcpa.2014.12.013. 25680848 10.1016/j.jcpa.2014.12.013

[12] Ueda K, Kawai T, Senoo H, Shimizu A, Ishiko A, Nagata M. Histopathological and electron microscopic study in dogs with patellar luxation and skin hyperextensibility. J Vet Med Sci. 2018;80(8):1309–16. 10.1292/jvms.18-0115. 29984735 10.1292/jvms.18-0115 PMC6115261

